# First-Year Healthcare Resource Utilization Costs of Five Major Cancers in Japan

**DOI:** 10.3390/ijerph18189447

**Published:** 2021-09-07

**Authors:** Tomone Watanabe, Rei Goto, Yoko Yamamoto, Yuichi Ichinose, Takahiro Higashi

**Affiliations:** 1 Division of Health Services Research, National Cancer Center, Tokyo 104-0045, Japan; yokyamam@ncc.go.jp (Y.Y.); yuichino@ncc.go.jp (Y.I.); thigashi@ncc.go.jp (T.H.); 2 Department of Social Medicine, Graduate School of Medicine, University of Tokyo, Tokyo 113-8654, Japan; 3 Graduate School of Business Administration, Keio University, Yokohama 223-8526, Japan; reigoto@kbs.keio.ac.jp

**Keywords:** healthcare costs, major cancers, Japan

## Abstract

Reports on the expenditure of cancer treatments per patient using comprehensive data remain unavailable in Japan. This study aimed to use Japan’s cancer registry data and health service utilization data for evaluating the disease-specific, per-patient costs of five major cancers—stomach, lung, colorectal, liver, and breast cancers. We used a database linking the 2017 data from a hospital-based cancer registry and the health service utilization data from the Diagnosis Procedure Combination survey. All patients who started their first treatment course at each hospital were included. The costs were calculated using the total volume of the health services provided and the unit fee information included in the data. We analyzed 304,698 patients. Lung cancer had the highest healthcare cost per-patient for the first year of diagnosis and the longest median hospitalization duration. Conversely, breast cancer showed the lowest cost and the shortest median hospitalization duration. However, in the first month after diagnosis, colorectal cancer showed the highest cost. Subsequently, the gaps between the costs of the five common cancers drastically diminished. The cancer type having the longest hospitalization duration had the highest overall healthcare resource utilization costs. This information is essential for care planning and research studies.

## 1. Introduction

The burden of cancer is continuously increasing. In 2018, around 16.8 million patients were newly diagnosed with cancer worldwide, leading to 9.6 million deaths, which have been constantly rising each year [1]. Healthcare resource utilization costs for cancer is increasing sharply, especially in high-income countries. For instance, in the United States, the healthcare resource utilization costs for 17 cancers reached 124.57 billion USD in 2010 and is expected to increase by 39% over the next decade [2]. The rising costs present a huge challenge for policymakers with respect to adequate resource management, which incorporates a comprehensive evaluation of resources used [3,4].

Likewise, Japan has increased incidence rates, causing a rapid surge in health expenditures, and an estimated 40 billion JPY is spent annually on cancer care [5,6,7]. This increment is possibly attributable to an increase in the number of patients resulting from the country’s high aging population, and technological advances producing more expensive tests and treatments into the market. To develop measures necessary for appropriate resource allocation, policymakers need accurate information on how the resources are used to treat major diseases. These data will aid in establishing evidence-based policymaking in order to address the prospect of healthcare expenditures that are robust to change.

However, in Japan, no studies have used comprehensive data to investigate and report the detailed expenditures per patient. Therefore, this study aimed to describe the disease-specific, per patient costs of five major cancers, namely, stomach, lung, colorectal, liver, and breast cancers, using the nationwide cancer registry data and health service utilization data in Japan. Cancer control in Japan has long focused on these five cancers, which have high incidence or mortality rates [5]. This study also sought to examine the patterns of resources used, such as hospitalization duration or the number of hospital visits, to provide policymakers the information of service utilization patterns necessary for planning healthcare systems.

## 2. Materials and Methods

### 2.1. Data Sources

We used a database that linked the data from a nationwide hospital-based cancer registry (HBCR) [8,9] and health service utilization data from the Diagnosis Procedure Combination (DPC) survey. The HBCR is a compulsory cancer incidence reporting system for all designated cancer care hospitals and non-designated hospitals that play similar roles in their local communities. The HBCR data include the clinical and pathological stages, tumor–node–metastasis (TNM) classifications (UICC 7th edition), tumor location, and histopathology according to the International Classification of Diseases Oncology 3rd edition (ICD-O-3). Using the database, we identified patients diagnosed with the five aforementioned cancers (ICD-O morphology codes: C16, C18–20, C34, C50, and C22) and included them in the analyses. Meanwhile, the DPC survey data contain information on all health services provided. Unlike DRG based per case payment system, the DPC based payment is a grouping system used to determine the amount of per day health insurance reimbursement to hospitals [10]. Additionally, its survey data contain the equivalent data for fee-for-service claims that register individual diagnostic tests, images, and procedures as well as the pharmacy claims for the prescription drugs. They also include the dates and unit costs of the services based on the national fee-schedule. In Japan, the health services are reimbursed to the providers on a fee-for-service basis, and the national fee-schedule define the amount of reimbursement for all individual health services ranging from physicians’ services to laboratory tests, imaging services, drugs as well as prescription drugs [10,11]. Each price is set to reflect the cost structure of the production of each health service. The amount of reimbursement under fee-for-service system is widely used as the proxy of costs, from both inpatient and outpatient settings [12]. The utilization data along with HBCR data collected at each hospital were submitted to National Cancer Center for data linkage. The selected time period for DPC data included all treatments performed for cancers diagnosed in 2017 (from 1st January to 31st December). In order to include all costs during a year from diagnosis, the DPC data from January 2017 to December 2018 were used for this study. The details of data collection process are described elsewhere [9].

### 2.2. Statistical Analysis

We calculated the average and median healthcare resource utilization (HCRU) costs related to cancer care, number of hospitalizations and outpatient visits, and hospitalization duration per patient during the first year after the diagnosis date. All patients who started their first treatment course at each hospital were included. The costs were calculated using the total volume of the health services provided and the unit fee information found in the data. Costs were calculated as fee-for-service in this analysis in order to present the resources used for each cancer. In other words, we added each unit cost for each service provided. The service fees represent the service costs, considering that the unit fee schedule is identified to reflect the resources used in Japanese average healthcare providers. The total cost for outpatient care and inpatient care within a year per patient was calculated. The total cost for each cancer was then stratified according to patients’ age, examining the amount of costs incurred per age stratum. All analysis data were confined to the first 12 months after the diagnosis date. Thus, we excluded all data obtained beyond the period or the cases that did not have the diagnosis date. If the discharge date exceeded 365 days after the diagnosis date, we included the cost incurred until a year after the diagnosis date.

The cost variation across the individual cases was analyzed using a multilevel mixed effects linear model, separating out the statistical variance of cancer care costs from the grand mean within the hospital and individual cluster for each cancer type.

All statistical data were analyzed using Stata version 14.2 (StataCorp LP, College Station, TX, USA). The institutional review board of the National Cancer Center in Japan approved our study protocol.

## 3. Results

The study analyzed 304,698 patients. Table 1 shows the patients’ demographic information. For each cancer, the results are presented as patients’ age and their clinical stage (I–IV).

### 3.1. Costs in the First Year of Diagnosis

Table 2 summarizes the distribution of the first-year costs per patient for the five common cancers. Lung cancer obtained the highest median overall costs per patient (2,508,789 JPY), while breast cancer showed the lowest (1,559,274 JPY). According to clinical stage, stage III cancers exhibited the highest median inpatient costs, except for colorectal cancer for which the highest inpatient cost was for stage IV. Stage III cancers also showed the highest outpatient median costs, except for liver and colorectal cancers. According to age group, patients in their 60s and 70s had the highest cancer care costs for all cancers. However, unlike other cancers, more than 50% of breast cancer care costs were consumed by patients below 60 years old.

**Figure 1:**
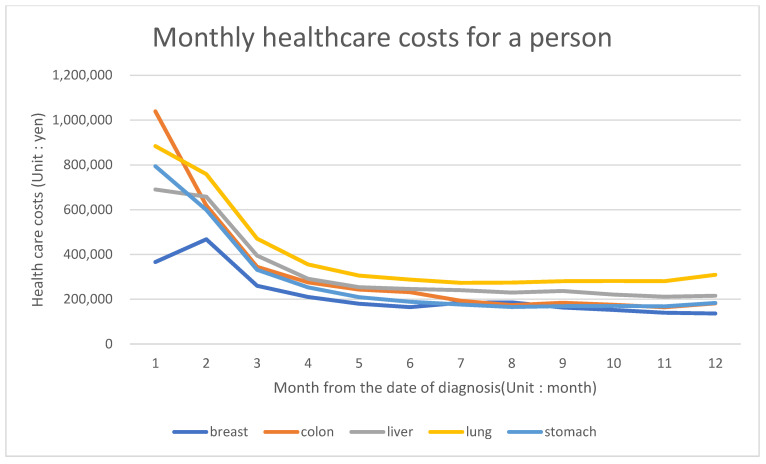
Monthly healthcare cost of each of the five major cancers per-patient.

**Figure 2:**
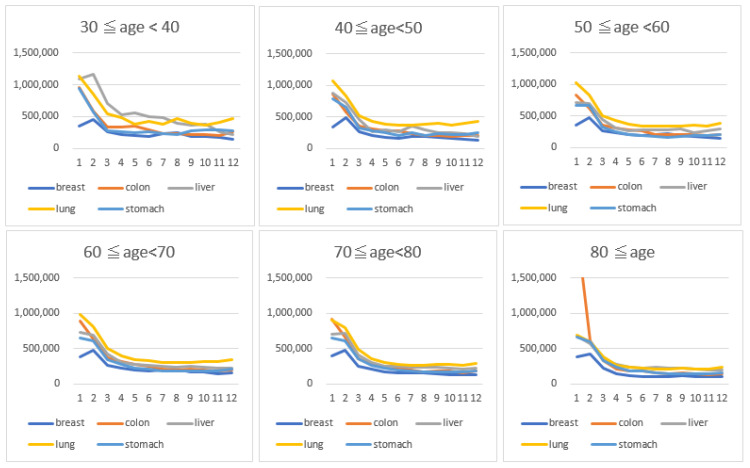
Monthly healthcare cost of each of the five major cancers per-patient stratified by age.

Figure 1 illustrates the trend of average monthly cancer care costs per patient. Except for the first month, the lung cancer costs were highest throughout the year, although the gap drastically diminished after 2 months from diagnosis. The costs for all cancers, excluding breast cancer, were highest at the first month after diagnosis, but on the second month, breast cancer showed the highest. Although breast cancer generally had the lowest cancer care costs up until the sixth month threshold, it leveled with the colorectal and gastric cancer costs thereafter. When stratified by age, the cost trend was fairly similar across patients aged 40–79 years. Meanwhile, the treatment cost for liver cancer was relatively higher in patients in their 30s, and that for colorectal cancer in the first month was conspicuously the highest in patients aged 80 or above (Figure 2).

### 3.2. Number of Outpatient Visits in a Year

Table 3 presents the trend for the number of outpatient visits and hospitalization. Breast cancer showed the highest median number of outpatient visits (25 visits), while other cancers obtained less than 15 visits. In addition, the median number of hospitalizations was within the range of 1–3. Together with other factors, outpatient visits impacted the total number of hospital visits than the number of hospitalizations. For most cancers, patients in stage II or stage III visit hospitals most frequently.

### 3.3. Average Hospitalization Duration

Table 4 shows the distribution of hospitalization duration. The median hospitalization duration was longest in lung cancer (22 days) and shortest in breast cancer (9 days). Stages III and IV marked the longest hospitalization duration for all cancers.

### 3.4. Statistical Variance of Cancer Care Costs

The individual monthly cancer care costs from the diagnosis were modeled as fixed parameters, and the facilities and individual patients as random parameters (Table 5). We hypothesized that the patient-defined statistical variance is always greater than the facility-defined variance. The standard deviation (SD) of hospital level cancer care costs from the grand mean was smaller than that of individual level cancer care costs from the hospital mean, except for breast cancer. Colorectal cancer had the greatest SD of hospital level cancer care costs from the grand mean, while lung cancer showed the smallest. Colorectal cancer also exhibited the greatest SD of individual-level cancer care costs from the hospital mean, while breast cancer obtained the smallest.

## 4. Discussion

In summary, our study revealed that the treatment costs vary across major cancers in Japan. Lung cancer obtained the highest overall costs for the first year after diagnosis and the longest median hospitalization duration (22 days). In contrast, breast cancer showed the lowest costs and the shortest hospitalization duration. In the first month after diagnosis, colorectal cancer showed the highest costs. Nevertheless, the gaps between the costs among these five cancers drastically diminished in the following months. Furthermore, the age-stratified cancer care costs in the first year of diagnosis were relatively the same across the age strata, except for patients in their 30s and those aged 80 years and above. Additionally, patient defined statistical variance was always greater than facility defined statistical variance, indicating that the costs were determined by the care provided for each patient.

The overall trend of costs for the first year agrees with those in other studies conducted in the US and elsewhere [2,13,14,15]. Colorectal cancer is reportedly the most expensive in the initial treatment phase. The initial and terminal phase costs for colorectal cancer were previously discussed [2,16]. One of the greatest contributors to this phenomenon is inpatient costs such as the personalized use of drugs and more tests [17]. In Japan, although the costs incurred at the first month of diagnosis for colorectal cancer is similar to those for lung cancer, the costs for lung cancer are always slightly ahead of the curve. Although the incidence of colorectal cancer outnumbers that of lung cancer, lung cancer mortality remains to be higher [18]. As stage information as well as types of treatment in Table 1 shows, more patients with lung cancers are diagnosed at the later stage [19], possibly leading to higher costs. Japanese studies suggest less expensive ways of treating colorectal cancer, such as the use of KRAS test before a particular chemotherapy as opposed to KRAS preselection test; however, further studies are needed to ascertain its effect [17,20]. Moreover, greater hospital level statistical variances of cancer care costs, especially for colon cancer, may implicate practice variations across hospitals.

A longer hospitalization duration is associated with higher healthcare resource utilization costs [21]. Although Japan is well known for its relatively long hospitalization duration [22], no single solution can solve this long standing issue, considering that various aspects of the disease and social characteristics determine the hospitalization duration [23]. The nation’s aging population continues to rise; thus, the high cancer care costs incurred by prolonged hospitalization duration are expected to increase further. In addition, focusing on a holistic approach to care, such as promoting a smooth transition to home-based care, augmenting social resources and support, and providing team-based management of comorbidity, has become increasingly important to reduce the overall costs.

However, our study has several limitations. First, we failed to capture the details of care provided at other hospitals if a patient moves from one hospital to another, although such cases are uncommon, as revealed by other studies [24]. This lapse could still potentially underestimate the amount of medical care cost per-patient. Although cancer treatment costs involve not only medical expenses but also travel expenses and opportunity expenses, which are a forgone value of time for patients and their family, we only reported the medical care costs based on the national fee schedule, thereby potentially limiting the application of the findings. Nevertheless, the baseline raw data were well represented in our study and considered important, especially in determining the overall cancer care costs. In 2019, Japan introduced an official health technology assessment system, which suggests that conducting more economic evaluations of cancer care are necessary. In conducting such economic evaluations, individual medical care costs must be identified. Finally, we analyzed the absolute cancer care costs, not the incremental cost matched with those costs in patients without cancer. If the policy target is the extensive resource allocation to cancer care, comparison with the costs for noncancer diseases is required.

## 5. Conclusions

Using the nationwide database, we successfully described the healthcare resource utilization costs of five major cancers (stomach, lung, colorectal, liver, and breast cancers) and their trend after diagnosis. The cancer that had the longest hospitalization duration had the highest overall costs. The findings of this study provide information necessary for care planning and research studies. Such data should be monitored regularly, and the policymakers should contain the costs as efficient as possible.

## Figures and Tables

**Table 1:** Patient demographics (2017).

	Liver		Stomach		Colorectal		Lung		Breast	
*n*	15,808		69,695		90,912		70,264		58,019	
Mean age (SD)	72.7 (10.2)		72.2 (10.4)		70.2 (11.5)		71.7 (10.4)		60.6 (14.1)	
Stage (%)										
0	-		-	-	23,086	25.4	156	0.2	7584	13.1
I	7375	46.7	44,312	63.6	17,542	19.3	28,310	40.3	23,997	41.4
II	4156	26.3	6292	9	17,737	19.5	5708	8.1	17,320	29.9
III	2665	16.9	6935	10	17,905	19.7	11,753	16.7	5938	10.2
IV	1274	8.1	11,205	16.1	13,540	14.9	23,173	33	2965	5.1
unknown	338	2.1	951	1.4	1102	1.2	1164	1.7	215	0.4
Age group (%)										
0–29	17	0.1	66	0.1	138	0.2	50	0.1	219	0.4
30–39	49	0.3	497	0.7	862	1	312	0.4	2678	4.6
40–49	331	2.1	1785	2.6	4120	4.5	1681	2.4	12,939	22.3
50–59	1183	7.5	4919	7.1	9947	10.9	5268	7.5	11,685	20.1
60–69	3962	25.1	18,317	26.3	25,719	28.3	20,024	28.5	13,947	24
70–79	5886	37.2	26,431	37.9	30,353	33.4	27,856	39.6	10,780	18.6
80–	4380	27.7	17,680	25.4	19,773	21.8	15,073	21.5	5771	10
Types of treatment *										
Surgery	12,887	81.5	59,369	85.2	81,231	89.4	39,005	55.5	51,245	88.3
Chemotherapy	7106	45	17,929	25.7	27,848	30.6	32,065	45.6	19,826	34.2
Radiation	1199	7.6	3106	4.5	4507	5	16,746	23.8	21,262	36.6

* Data does not add up to 100% due to duplication.

**Table 2:** Mean healthcare resource utilization costs of the five major cancers within a year after diagnosis per-patient in Japan (upper row: median, lower row: mean, unit: Yen).

	Unit	0	I	II	III	IV	Unknown	All Stages	Total (Outpatient + Inpatient)
Liver (*n* = 15,808)	Outpatient		300,931	329,004	219,278	87,479	78,362	287,589	
			618,592	631,108	500,150	517,268	284,751	586,611	2,078,843
	Inpatient		1,452,499	1,819,571	2,159,867	1,304,000	1,113,121	1,623,739	2,671,490
			1,826,721	2,284,767	2,618,544	1,855,085	1,918,391	2,084,879	
Stomach (*n* = 69,695)	Outpatient		185,434	372,081	569,867	364,153	62,096	214,528	
			292,830	521,520	790,280	1,071,880	320,411	488,600	1,839,530
	Inpatient		918,689	2,011,916	2,290,073	1,876,265	916,390	1,509,190	2,421,138
			1,654,133	2,487,039	2,754,346	2,253,904	1,456,846	1,932,538	
Colorectal (*n* = 90,912)	Outpatient	93,885	204,391	242,377	702,285	921,884	40,656	222,396	
		230,635	323,130	441,633	852,293	1,691,874	408,588	631,870	2,058,540
	Inpatient	371,859	1,632,299	1,872,095	2,009,314	2,345,584	1,141,618	1,630,101	2,713,002
		728,364	1,849,647	2,361,973	3,198,947	2,875,019	1,668,914	2,081,132	
Lung (*n* = 70,264)	Outpatient	155,190	255,066	391,025	537,181	383,286	82,491	317,223	
		210,582	414,857	683,542	1,231,550	1,681,510	401,530	990,358	2,508,789
	Inpatient	1,700,838	1,899,493	2,432,563	2,518,769	1,910,023	937,621	1,979,050	3,366,480
		1,846,962	2,069,106	2,785,343	2,849,596	2,460,904	1,438,782	2,376,122	
Breast (*n* = 58,019)	Outpatient	233,410	590,118	809,373	1,367,112	1,313,672	265,459	647,501	
		386,271	728,104	1,076,681	1,537,909	2,239,234	800,032	947,850	1,559,274
	Inpatient	726,813	744,886	928,522	1,085,048	809,365	376,633	820,950	2,012,925
		919,149	970,355	1,133,881	1,353,685	1,253,190	676,353	1,065,075	

**Table 3:** Annual number of outpatient visits and hospitalization per-patient (upper: median, lower: mean).

	Unit	0	I	II	III	IV	Unknown	Total
Liver	Outpatient	-	12	13	9	3	4	12
			14	14	11	8	7	13
	Inpatient	-	2	2	2	1	1	2
			2	2	2	2	2	2
Stomach	Outpatient	-	10	15	18	12	3	11
			12	16	19	15	8	13
	Inpatient	-	1	1	2	2	1	1
			2	2	2	3	2	2
Colorectal	Outpatient	5	11	12	17	19	2	11
		8	13	14	18	19	8	14
	Inpatient	1	1	1	2	2	1	1
		1	2	2	2	3	2	2
Lung	Outpatient	8	12	17	19	11	4	13
		10	14	19	21	15	9	15
	Inpatient	1	1	2	3	2	1	2
		1	2	3	3	3	2	2
Breast	Outpatient	16	25	27	37	22	10	25
		21	26	30	37	24	16	27
	Inpatient	1	1	1	1	1	1	1
		1	1	2	2	1	1	1

**Table 4:** Hospitalization duration per-patient (upper: median, lower: mean).

	0	I	II	III	IV	Unknown	Total
Liver	-	17	23	34	28	20	21
		25	32	42	36	32	31
Stomach	-	12	23	29	34	22	16
		19	34	40	43	32	27
Colorectal	4	16	22	24	34	23	18
	9	23	31	33	43	33	26
Lung	10	12	28	46	37	19	22
	14	18	39	53	48	31	36
Breast	8	8	10	13	10	6	9
	10	11	14	19	23	13	13

**Table 5:** Statistical variance of cancer care cost using mixed method.

	Liver	Stomach	Colorectal	Lung	Breast
Grand mean	2,605,715	2,493,774	2,823,430	3,311,205	2,118,407
Facility level (SD)	904,880	2,589,735	4,077,456	485,880	2,015,123
Individual level (SD)	2,398,249	39,439,543	41,150,914	2,513,716	1,609,051

## Data Availability

The datasets generated during and/or analyzed during the current study are available from the corresponding author on request.
